# Metabolomic analysis reveals spermatozoa and seminal plasma differences between Duroc and Liang guang Small-spotted pig

**DOI:** 10.3389/fvets.2022.1078928

**Published:** 2023-01-06

**Authors:** Zhili Li, Jingshuai Sun, Kebiao Li, Jiali Qin, Yanmei Sun, Jianhua Zeng, Saeed El-Ashram, Yunxiang Zhao

**Affiliations:** ^1^College of Life Science and Engineering, Foshan University, Foshan, China; ^2^College of Animal Science, South China Agricultural University, Guangzhou, China; ^3^Guangxi Yangxiang Co., Ltd., Guigang, China; ^4^Guangdong YIHAO Food Co., Ltd., Guangzhou, China; ^5^Kafrelsheikh University, Kafr El-Sheikh, Egypt

**Keywords:** metabolomic, boar, spermatozoa, seminal plasma, Liang guang small-spotted pig

## Abstract

The Liang guang Small-spotted pig is a well-known Chinese indigenous pig that is valued for its exceptional meat quality. However, the Liang guang Small-spotted pig has a lower semen storage capacity, shorter storage time and worse semen quality compared to Duroc. Pig sperm used for artificial insemination (AI) loses part of vitality and quality when being stored in commercial solutions. Serious vitality losses and short shelf life of the semen are particularly prominent in Liang guang Small-spotted pig. In this study, the metabolites in seminal plasma and spermatozoa of Duroc and Liang guang Small-spotted pigs were identified using UHPLC–Q-TOF/MS technology. The findings indicated forty distinct metabolites concentrating on energy metabolic substrates and antioxidant capacity in Liang guang Small-spotted pig and Duroc seminal plasma, including D-Fructose, succinate, 2-dehydro-3-deoxy-d-gluconate, alanine betaine, citrate, carnitine, acetylcarnitine and so on. Seventeen different metabolites were explored, with a focus on glycerophospholipid metabolism in Liang guang Small-spotted pig and Duroc spermatozoa, primarily including glycerol 3-phosphate, acetylcarnitine, phosphatidylcholine (PC) 16:0/16:0, palmitoyl sphingomyelin, acetylcholine, choline, glycerophosphocholine, betaine, L-carnitine, creatinine and others. This study reveals the metabolite profile of spermatozoa and seminal plasma among different pig breeds and might be valuable for understanding the mechanisms that lead to sperm storage capacity. Metabolites involved in energy metabolism, antioxidant capacity and glycerophospholipid metabolism might be key to the poor sperm storage capacity in Liang guang Small-spotted pig.

## 1. Introduction

Artificial insemination (AI) is one of the most extensively utilized breeding techniques in today's pig husbandry (1). The quality of boar sperm is critical for the development of AI, which is determined by environmental and genetic (2). Boar semen extended in liquid form is used the same day or stored at 15–20 °C for 1–5 days waited for AIs (3). Boar sperm quality gradually degraded, always loses part of vitality and quality when being stored in commercial solutions. The Liang guang Small-spotted pig, also known as the Lu Chuan pig or Guang dong Small-spotted pig, is an obese breed with strong reproduction, a low growth rate, and good meat quality (4). Duroc has a number of complementing qualities, including a reduced fat rate, a higher growth rate, and a lower fertility rate. Although the Liang guang Small-spotted pig has superior reproductive performance, its sperm has lower storage capacity and semen quality than Duroc sperm. The molecular mechanisms underlying their semen differences are yet unknown.

Seminal plasma (SP) contains secretions from accessory sex glands and accounts for 95% of semen (5, 6). It assists in the maturation, motility, and fertilization of mammalian sperm, however, its role in sperm storage is still unknown. Seminal plasma was frequently diluted or eliminated before liquid storage and cryostorage. Adding the proper number of seminal plasma to frozen sperm had been proven to increase acrosome integrity and sperm quality in studies (3, 7). Previous studies had demonstrated that re-adding seminal plasma to isolated sperm during liquid storage of semen at 17 °C modulated sperm's ability to elicit *in vitro* capacitation and undergo acrosomal exocytosis (8). Mature mammalian spermatozoa are peculiar elongated and highly differentiated haploid cells, which carry the paternal genes that are silent in transcription and translation. They are carefully controlled by available proteins and post-translational modifications (9).

Metabolomics is a new technology that studies low molecular weight compounds to uncover the regulatory mechanisms of spermatozoa. It has been shown that sperm metabolites might control signaling pathways such as sperm motility, hyperactivation, and energy acquisition-related signaling directly or indirectly (10). In recent years, spermatozoa and seminal plasma metabolomics has been utilized mostly to identify biomarkers in human (11), as well as bulls (12, 13), boars (14). These metabolomics studies on mammalian sperm were mostly focused on identifying high or low fertility. Recently, the association between seminal plasma metabolites and sperm quality was investigated. Some metabolites, including leucine, taurine, and carnitine, related to spermatozoa's fluid storage capacity (15).

To further understand the differences in semen and identify differential metabolites, in this study, the computer-aided semen analysis system (CASA) was used to analyze sperm motility and the UHPLC–Q-TOF/MS technique was used to discover metabolic differences between the sperm and seminal plasma of Duroc and Liang guang Small-spotted pigs. And with a view of contributing to identify potential biomarkers for the poor storage capacity of Liang guang Small-spotted pigs.

## 2. Materials and methods

### 2.1. Semen samples

Duroc (*n* = 6) and Liang guang Small-spotted pigs (*n* = 6) aged from 18 to 24 months were raised on the same farm (Zhanjiang, Guangdong Province, China) and fed the same diet under the same conditions. All of the boars were maintained in separate pens and given free access to water. Semen samples were collected by the technician of the swine farm using the gloved-hand technique. The samples divided into two parts, one for the semen quality assessment and the other one for metabolomic analysis. The sperm-rich fraction was centrifuged for 10 min at 850 g at 17 °C after collection. The sperm samples were separated into two groups: Duroc sperm (DS) and Liang guang Small-spotted pig sperm (LS). The supernatants (seminal plasma) were obtained from the Duroc seminal plasma (DSP) and the Liang guang Small-spotted pig seminal plasma (LSP). The samples were stored at −80 °C prior to further processing for UHPLC-Q-TOF/MS analysis.

### 2.2. Sperm motility evaluation

A total of 36 ejaculates (18 ejaculates per breed) were collected from 12 AI-boars (three ejaculates per boar) during a 3-month period in spring (one ejaculate/per boar/ month). Semen collected during the 1st month was used for motility, progressive motility, acrosomal membrane integrity, mitochondrial membrane potential, reactive oxygen species, and membrane integrity. Semen collected in the second 2 months was used for analysis of motility and progressive motility only. The computer-aided semen analysis system (CASA; IMV Technologies, France) was used to analyze sperm motility (at days 1, 3, and 5, respectively). The semen was diluted with commercial diluent and stored at 17 °C. Before sperm evaluation, samples were incubated at 37 °C for 10 min and then 10 μL was added to the depth slide. More than 4 non-consecutive fields (minimum 1,000 spermatozoa) were analyzed by CASA and total motility and progressive motility were recorded. The CASA system was set according to the following cut-off values: frames acquired, 30; frame rate, 60 Hz; motility, 5 μm/s; temperature, 37 °C. Progressive motile sperm were defined by values of average path velocity >45 μm/s and straightness >45%.

### 2.3. Flow cytometric evaluation

Semen samples were analyzed using a Guava EasyCyte flow cytometer (Merck Millipore MA, USA) equipped with 488 nm blue laser. Three fluorescence intensity channels were used with following characteristics: green fluorescence (525/30 nm); yellow fluorescence (583/26 nm); red fluorescence (655/50 nm). For all evaluations, a gate was created with the Side Scatter and Forward Scatter plot to consider only sperm. Flow cytometry was performed over 10,000 acquired events with InCyte software. Manual compensation was performed in all experiments and unstained cells were used as control.

Sperm reactive oxygen species (ROS) level was estimated using procedures described by Zhu et al. (16). The DCFH-DA assay was employed to determine the intracellular ROS, the intracellular DCFH-DA was deesterified to dichlorodihydrofluorescein which is oxidized by ROS to produce the fluorescent compound dichlorofluorescein (DCFH-DA; Beyotime, China). Samples were incubated at 37 °C utilizing 10 μM DCFH-DA for 30 min before analysis in the flow cytometer. The analysis was performed using a 488 nm excitation blue laser and emission detection at 525 nm for FITC. For each sample, three replicates were performed.

There was estimation of the sperm mitochondrial membrane potentials using JC-1 (JC-1; Beyotime, China) according to the manufacturer's protocol. Briefly, the sperm samples were incubated with JC-1 working solution for 30 min at 37 °C, protected from light. Then the sperm concentration was re-suspended to 2 × 10^7^/ml after washing twice with PBS. The lower mitochondrial membrane potentials were observed using FL1 (525/30 nm) for fluorescence signals, while higher mitochondrial membrane potentials were observed using FL2 (583/26 nm). For each sample, three replicates were performed.

Membrane integrity were assessed by LIVE/DEAD Sperm Viability Kit (Invitrogen TH, Thermo Fisher Scientific, USA). Briefly, sperm samples were incubated with SYBR 14 in the dark for 10 min at 37 °C and washed twice with pbs, followed by PI staining and incubation at 37 °C for 10 min. For the evaluation of plasma membrane integrity, F1 (525/30 nm) and F3 (655/50 nm) were used to detect SYBR and PI, respectively. Events with SYBR-14 positivity were considered to have membrane integrity, while those with PI positivity or with co-expression (moribund sperm) were considered to have membrane damage. For each sample, three replicates were performed.

The integrity of sperm acrosomes was evaluated using fluorescein isothiocyanate-labeled peanut (*Arachis hypogaea*) agglutinin (FITC-PNA; Sigma, USA) staining. Briefly, sperm samples were incubated sequentially with FITC-PSA and PI at 37 °C for 10 min. For the evaluation of integrity of sperm acrosomes, F1 (525/30 nm) and F3 (655/50 nm) were used to detect PSA and PI, respectively. For each sample, three replicates were performed.

### 2.4. Metabolite extraction

A total of 20 samples including 10 sperm and 10 Seminal plasma from 10 boars (five boars per breed) were used for metabolite extraction. The samples were added to a premix containing an internal target (V methanol: V acetonitrile: V water = 2:2:1). After sonication at a low temperature for 30 min, the sample was left to stand at −20 °C for 10 min and centrifuged at 14,000 g for 20 min at 4 °C. The supernatant was dried under a vacuum, and 100 μL of aqueous acetonitrile (acetonitrile: water = 1:1, v/v) was added for mass spectrometry and then centrifuged at 14,000 g for 15 min at 4 °C. The supernatant was finally taken into the sample for the UHPLC-QTOF-MS analysis.

### 2.5. UHPLC-QTOF/MS conditions

A UHPLC (1,290 Infinity LC, Agilent Technologies) was used in conjunction with a quadrupole time-of-flight analyzer (AB Sciex TripleTOF 6600). The chromatographic mobile phase was made up of (A) 25 mM ammonium acetate and 25 mM ammonium hydroxide in water and (B) acetonitrile. The elution gradient was as follows: 0 min, 95% B; 0.5 min, 95% B; 7 min, 65% B; 8 min, 40% B; 9 min, 40% B; 9.1 min, 95% B; 12 min, 95% B. The Triple TOF 6600 mass spectrometer (AB SCIEX) was used for MS analysis with electrospray ionization (ESI) in positive and negative ion modes, respectively. The ESI source conditions were set as follows: Ion source gas 1 is set to 60 psi, Ion source gas 2 is set to 60 psi, curtain gas is set to 30 psi, source temperature is set to 600 °C, and Ion Spray Voltage Floating (ISVF) is set to 5,000 volts in positive or negative mode, respectively.

### 2.6. Data processing and annotation

ProteoWizard converted the raw data in Wiff format to mzXML format, and then XCMS software was used for peak alignment, retention time correction, and peak area extraction. Peak alignment, retention time correction, and peak area extraction were then performed on the data using XCMS software. The XCMS data were first used to identify metabolite structures, then for data preprocessing, and finally for data quality assessment. The XCMS data were identified first through metabolite structure identification and data preprocessing, then through experimental data quality evaluation, and finally through data analysis. In addition, to investigate the underlying metabolic pathways, the Kyoto Encyclopedia of Genes and Genomes (KEGG) pathway database (https://www.kegg.jp/kegg/) was used. Ipath3 was used for data visualization (17).

### 2.7. Statistical analysis

Following sum-normalization, the data were analyzed with the R package (ropls), which performed multivariate data analysis, including Pareto-scaled principal component analysis (PCA) and orthogonal partial least-squares discriminant analysis (OPLS-DA). The model's robustness was assessed using 7-fold cross-validation and response permutation testing. The variable importance in the projection (VIP) value of each variable in the OPLS-DA model was calculated to indicate its contribution to the classification. The correlation between two variables was determined using Pearson's correlation analysis. Changes in sperm motility were assessed using repeated measures analysis of variance, while semen quality was assessed using one-way analysis of variance.

## 3. Results

### 3.1. Sperm assessments

**Figure 1:**
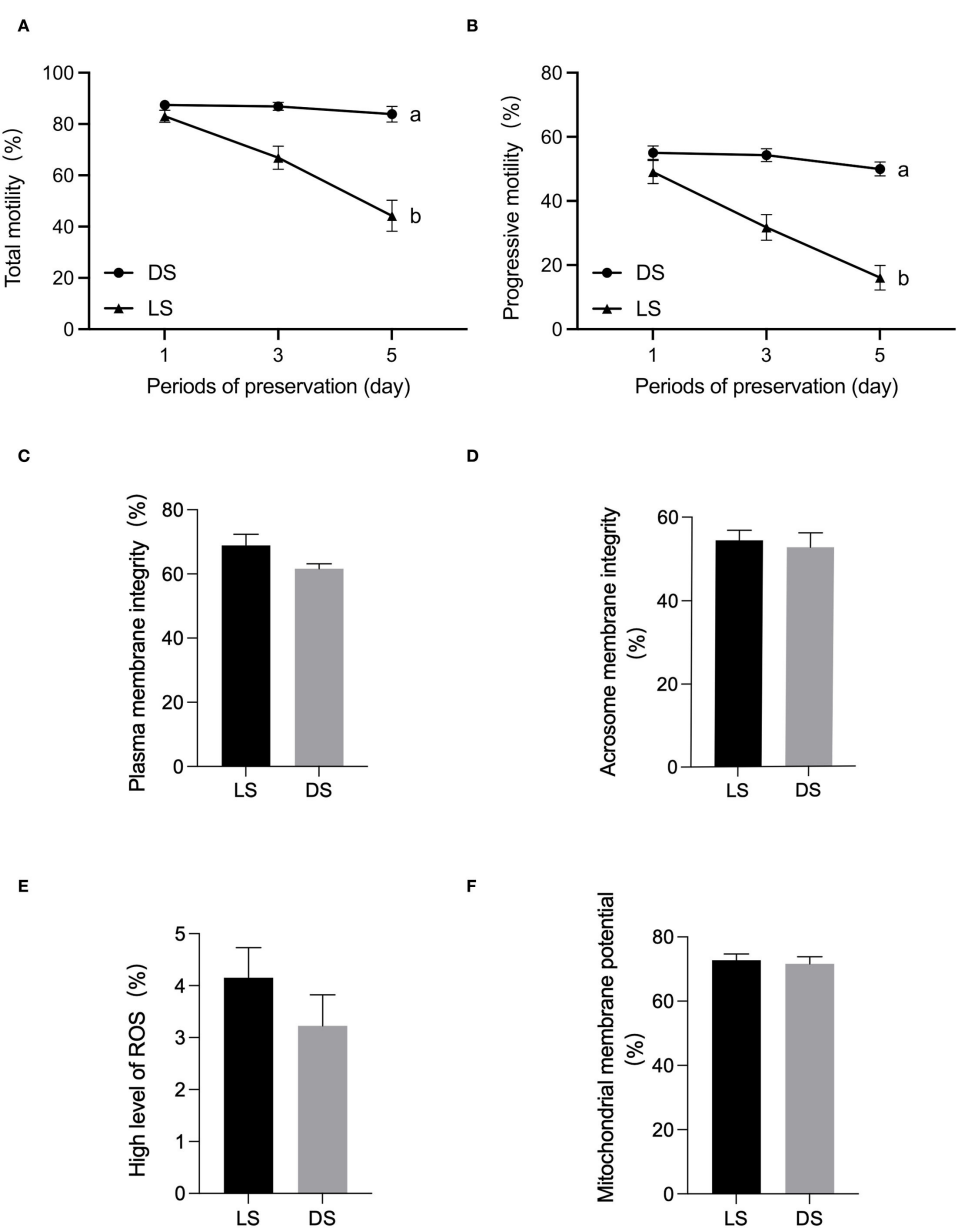
Assessment of semen quality parameters between Duroc and Liang guang Small-spotted pig. **(A)** Sperm total motility parameter was evaluated at days 1, 3, and 5 of semen preservation, respectively, **(B)** sperm progressive motility, **(C)** plasma membrane integrity, **(D)** acrosomal membrane integrity, **(E)** reactive oxygen species, and **(F)** mitochondrial membrane potential were assessed on day 1. Date are presented as the mean ± SEM, and different letters indicate significant differences; unmarked means the difference is not significant.

There was a tendency to lower levels of both total motility, progressive motility after 5 days in LS and DS. Significant differences in sperm total motility and progressive motility were observed between the LS and DS after 5 days (*P* < 0.05) (Figure 1). Furthermore, no significant differences were found between the two groups in terms of acrosomal membrane integrity, mitochondrial membrane potential, reactive oxygen species, or membrane integrity (*P* > 0.05) (Figure 1).

### 3.2. Metabolomic analysis based on UHPLC-qTOF-MS technology

**Figure 2:**
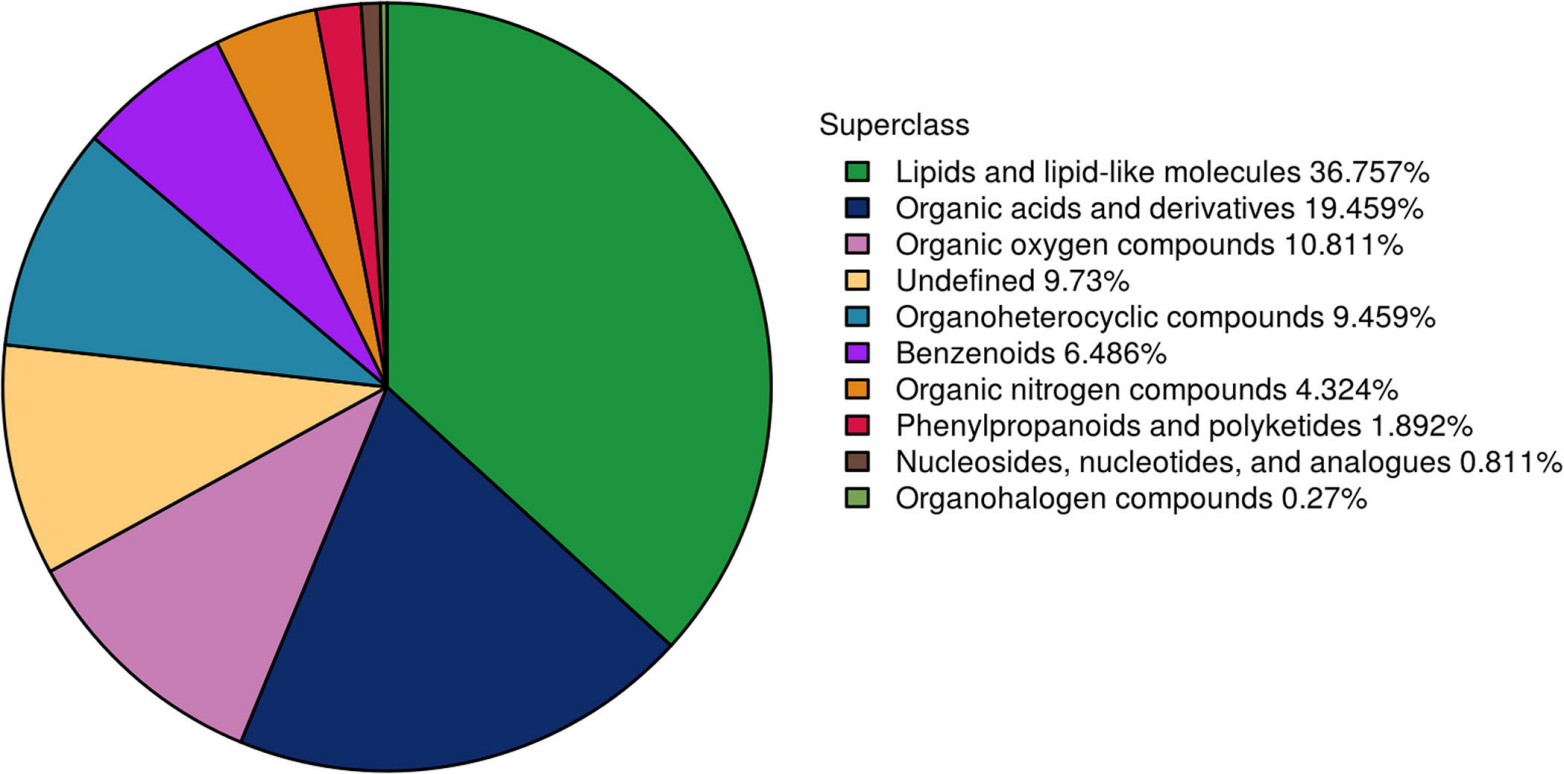
Presentation of the metabolomic differences.

To further understand the differences in semen between the two breeds and to reveal the reasons for the poor storage capacity of LS, the UHPLC–Q-TOF/MS technique was used to discover metabolic differences between the sperm and seminal plasma of Duroc and Liang guang Small-spotted pigs. Three hundred and seventy metabolites were identified in spermatozoa and seminal plasma samples, including 201 metabolites detected in the positive ion mode and 169 metabolites detected in the negative ion mode. Metabolites were identified and categorized according to their major chemical classes, including lipids and lipid-like molecules, organic acids and derivatives, organic oxygen compounds, undefined, organoheterocyclic compounds, Benzenoids, organic nitrogen compounds, phenylpropanoids and polyketides, nucleosides, nucleotides, and analogs, and organohalogen compounds (Figure 2).

### 3.3. Multivariate statistical analysis

**Figure 3:**
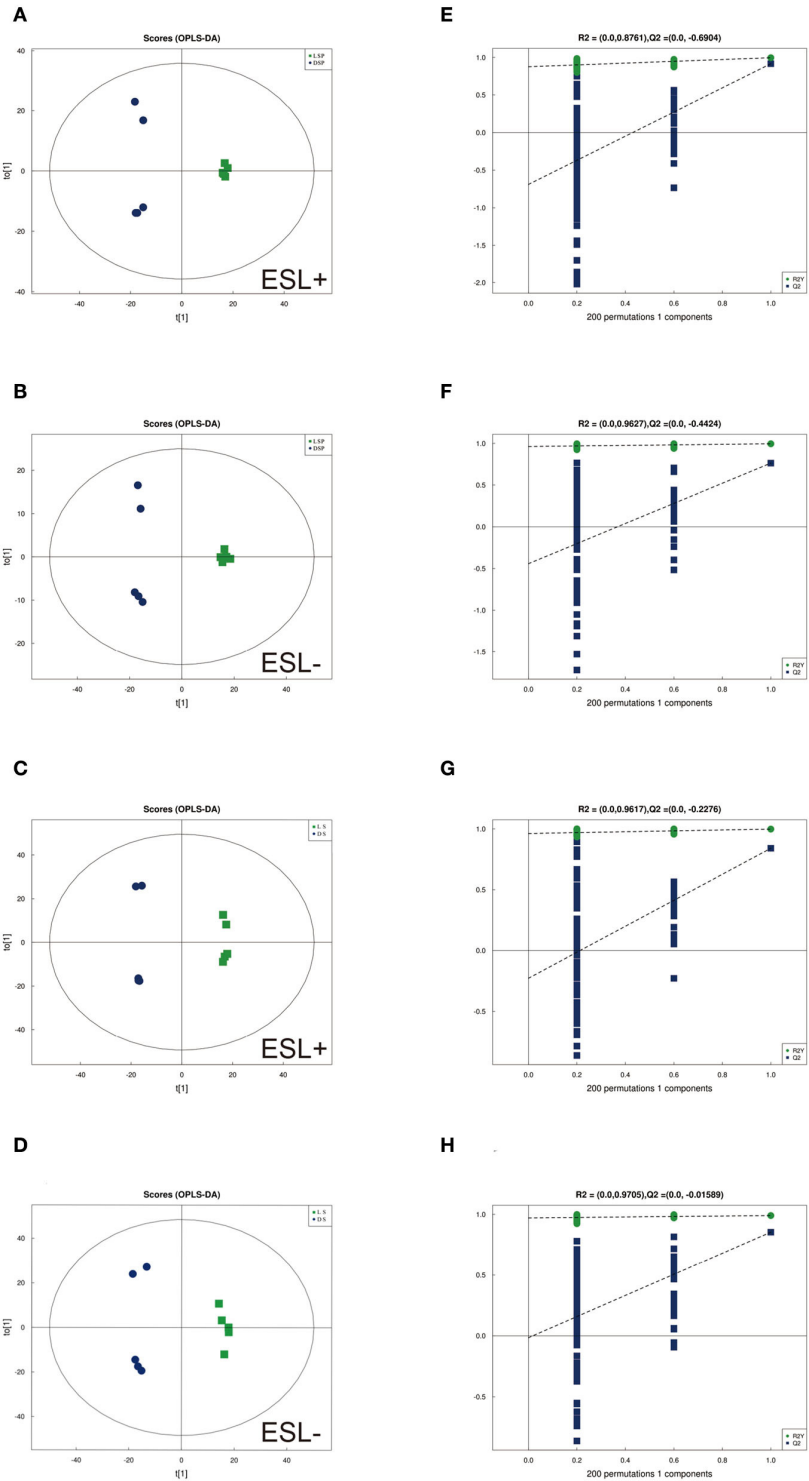
OPLS-DA scores plots based on sperm and seminal plasma samples showing the correlations between the DSP, LSP, DS and LS group in both positive and negative modes (*n* = 5 per group). **(A, B)** Seminal plasma scores plots of **(A)** R2y = 99%, Q2 = 0.92 (positive). **(B)** R2y = 99%, Q2 = 0.76 (negative). **(C, D)** Sperm scores plots of **(C)** R2y = 99%, Q2 = 0.84 (positive). **(D)** R2y = 99%, Q2 = 0.85 (negative). **(E–H)** Corresponding statistical validation of OPLS-DA models by permutation analyses.

**Figure 4:**
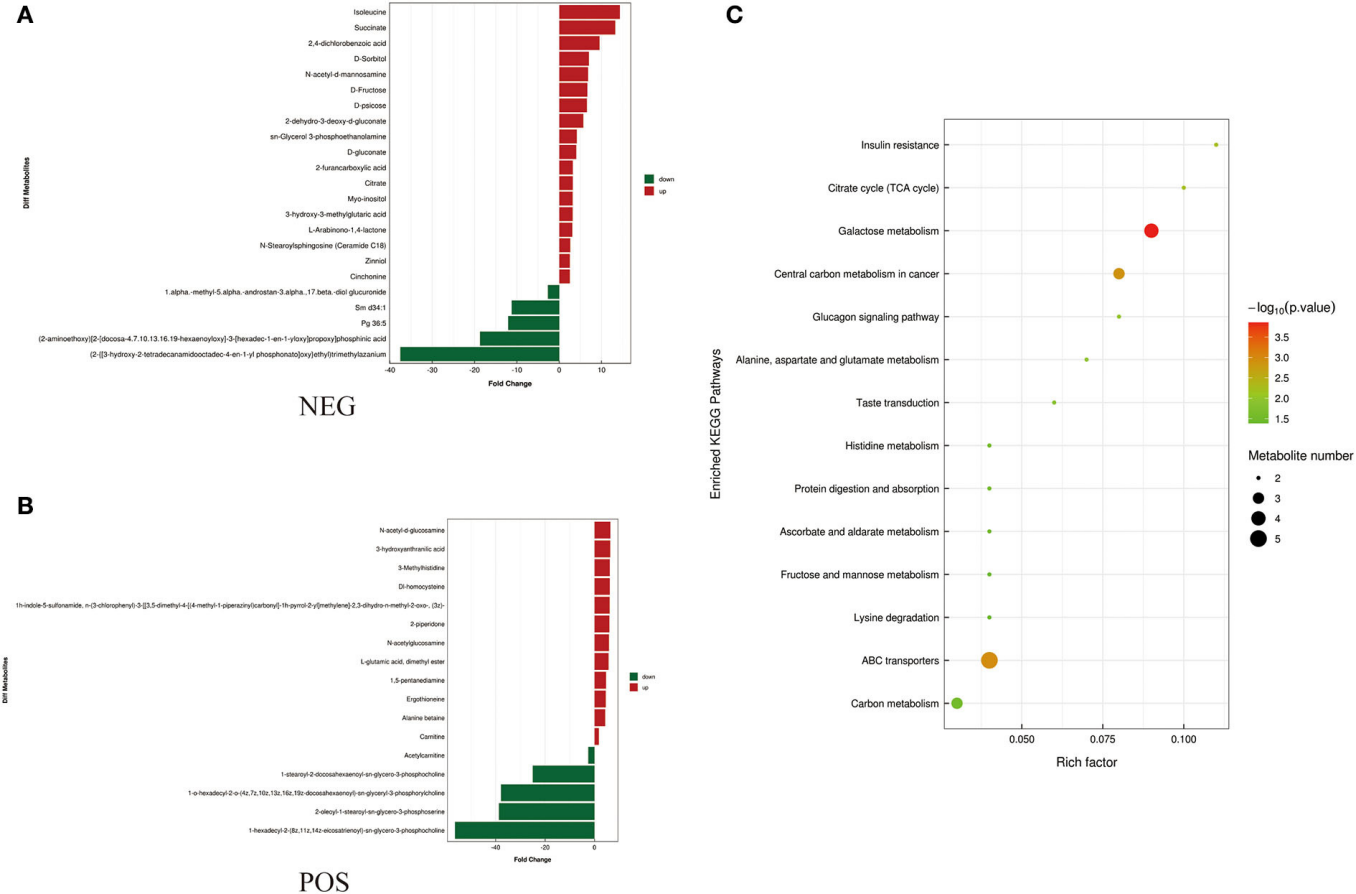
Metabolites identified in seminal plasma by OPLS-DA model VIP > 1. **(A, B)** Was detected in negative and positive ions mode respectively. **(C)** KEGG analysis of the differential metabolites in seminal plasma of Duroc and Liang guang Small-spotted pig. The bubble size indicates the influencing factor in the topological analysis; the bubble color represents the *P*-value of enrichment analysis.

**Figure 5:**
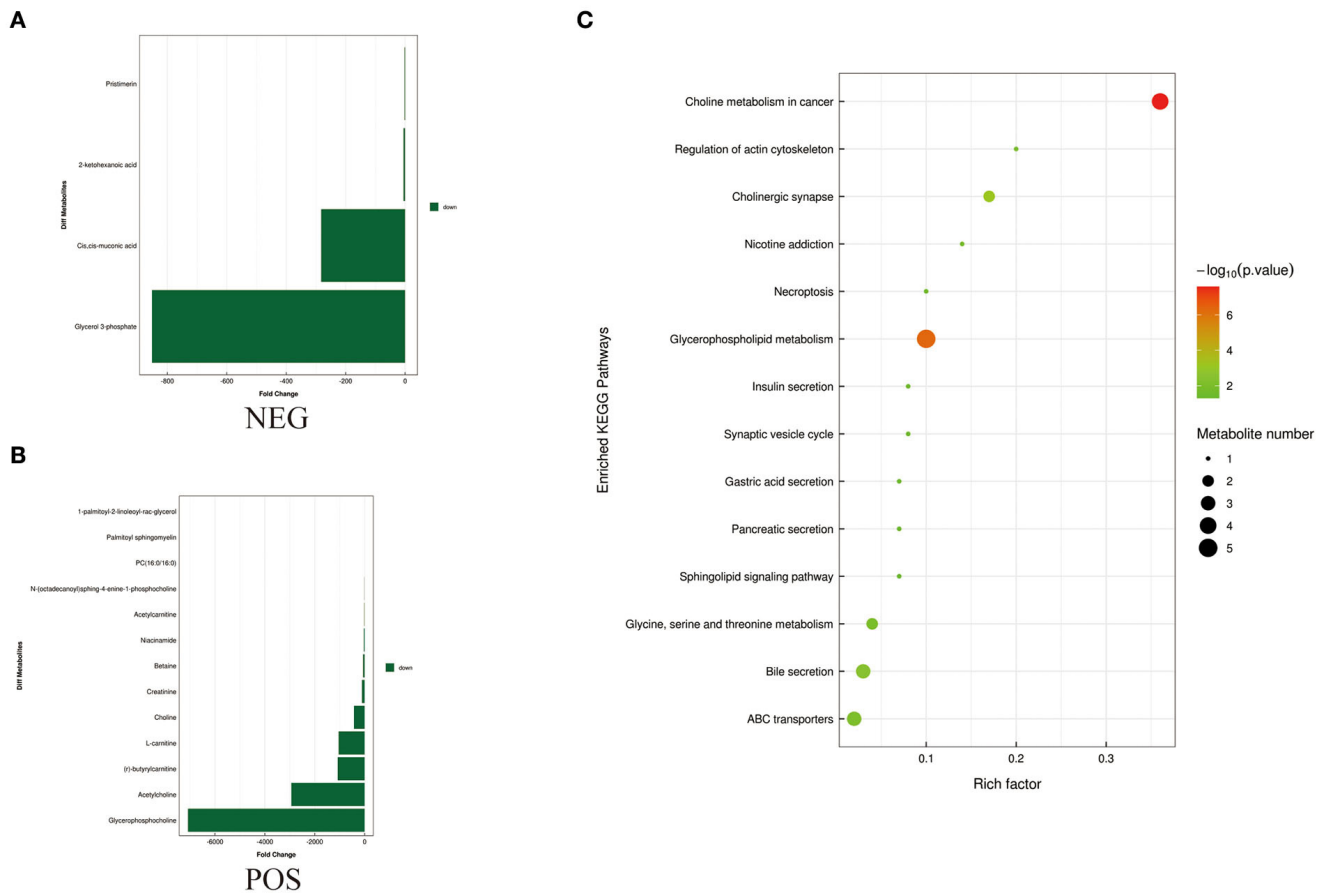
Metabolites identified in sperm by OPLS-DA model VIP > 1. **(A, B)** Was detected in negative and positive ions mode, respectively. **(C)** KEGG analysis of the differential metabolites in sperm of Duroc and Liang guang Small-spotted pig. The bubble size indicates the influencing factor in the topological analysis; the bubble color represents the *P*-value of enrichment analysis.

To identify differences in semen between the two pig breeds, the OPLS-DA model was applied to classify the LS vs. DS and the LSP vs. DSP, respectively. In the OPLS-DA scoring plot, there was a clear separation between the groups in the positive and negative ion modes (Figures 3A–D). To avoid over-fitting of the supervised model during the modeling process, the model was tested using the permutation test to ensure its validity. The results showed that the original model's overall R2Y and Q2 were higher than those of the model constructed by the replacement test, indicating that the original model grouping was not over-fitted (Figures 3E–H). On the basis of the OPLS-DA results, 17 and 40 significantly different metabolites (SDMs) were identified (VIP > 1, *P* < 0.05) in the LS vs. DS group and the LSP vs. DSP group, respectively (Figures 4, 5, Supplementary Table 1).

### 3.4. Identification of potential biomarkers and metabolic pathways

**Figure 6:**
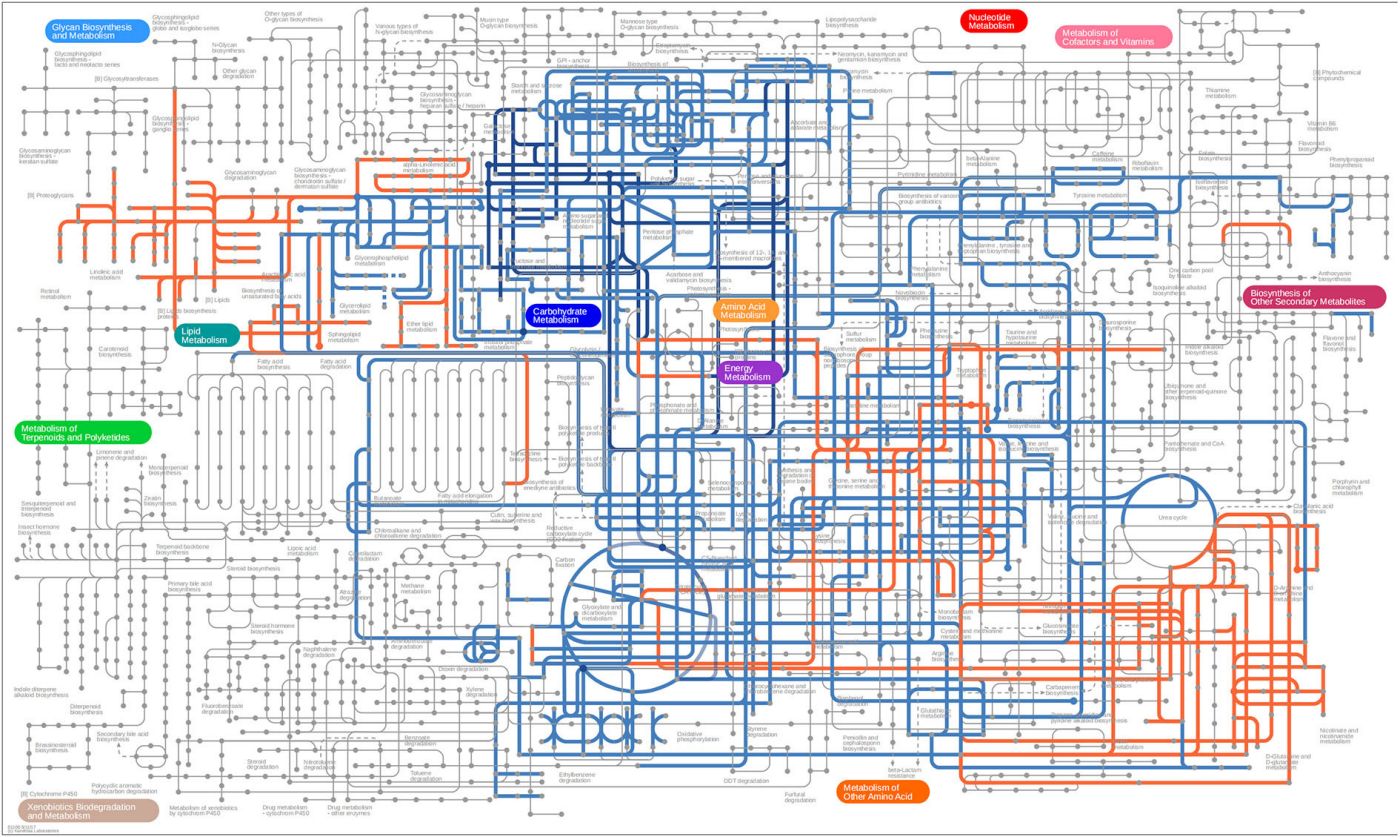
KEGG network annotated using iPath 3. Blue and orange represent the pathway analysis of differential metabolites in seminal plasma and sperm, respectively.

Differential metabolites screened by positive and negative ion patterns were combined and then performed KEGG pathway annotation. The results of KEGG showed that the differential metabolites in seminal plasma of the two pig breeds are mainly involved in the following metabolic pathways: metabolic pathways, ABC transporters, galactose metabolism, carbon metabolism, central carbon metabolism in cancer, citrate cycle (TCA cycle), alanine, aspartate, and glutamate metabolism, lysine degradation, glyoxylate and dicarboxylate metabolism, glucagon signaling pathway, fructose and mannose metabolism, taste transduction, Insulin resistance, amino sugar and nucleotide sugar metabolism (Figure 4C). In addition, the differential metabolites in the spermatozoa of the two pig breeds were mainly involved in the following metabolic pathways: glycerophospholipid metabolism, cholinergic synapse, bile secretion, ABC transporters, glycine, serine, and threonine metabolism, actin cytoskeleton regulation, nicotine addiction necroptosis, synaptic vesicle cycle, insulin secretion, gastric acid secretion, sphingolipid signaling pathway, and pancreatic secretion (Figure 5C). The annotation of KEGG results were further visualized and revealed that the distribution of differential metabolite functions in spermatozoa were more dispersed, while those in seminal plasma were mainly concentrated in energy metabolism (Figure 6).

## 4. Discussion

Sperm motility is one of the most intuitive and effective ways to assess the quality of sperm. Both breeds showed a decreasing trend in sperm motility but the sperm from Liang guang Small-spotted pig had more difficulty in preserving motility. The initial assessment of traditional sperm functional parameters such as acrosomal membrane integrity, mitochondrial membrane potential, reactive oxygen species, and membrane integrity were unable to reveal the semen characteristics of both breeds. Metabolomics has often been used in assessing sperm function. Therefore, the metabolites in the seminal plasma and spermatozoa of Duroc and Liang guang Small-spotted pigs were identified using UHPLC–Q-TOF/MS technology in this study.

Numerous antioxidant metabolites were identified in semen and some of them, such as ergothioneine, acetylcarnitine, inositol, and citrate, were found to be substantially different between the two pig breeds in this study. Interestingly, there was no significant differences in ROS levels in the initial evaluation of sperm, suggesting that antioxidant species in semen, especially in seminal plasma, may play a role in dynamic changes during sperm storage. An increase in reactive oxygen species and a reduction in antioxidants were detected when sperm samples were kept and refrigerated for a length time (18, 19). Antioxidants were abundant in semen, which helped to reduce oxidative damage (20). In a recent study, metabolomics of freeze-resistant pig semen has also highlighted the importance of antioxidant metabolites (21). Ergothioneine, which was significant difference between the two pig breeds, is an antioxidant that may be acquired from food and stored as an intracellular component in the blood and tissues (22, 23). In addition, high concentrations of ergothioneine were found in the seminal plasma of boars (23) and horses (24). Ergothioneine was also used as a semen diluent additive for the cryostorage of sperm in poultry (25), ram (26) due to its excellent antioxidant properties. Ergothioneine had been found in studies to increase sperm quality by enhancing sperm membrane function and protecting sperm DNA integrity (26).

Carnitine was discovered in high concentrations in the mammalian epididymis and spermatozoa as an antioxidant and energy source. Translocating fatty acids into the mitochondria for oxidation to produce ATP reduces the availability of lipid peroxidation. L-acetylcarnitine, the active form of carnitine, protects mitochondria from metabolic toxins by acting as an antioxidant. Carnitine had also been shown to protect the activity of pyruvate dehydrogenase, a mitochondrial respiration enzyme that traps excess mitochondrial acetyl-CoA as acetyl-L-carnitine (27, 28). Carnitine and L-carnitine supplementation in semen diluents had been shown to improve semen quality in bovine (29), boar (30), and horse (31), including increasing spermatozoa bound to the zona pellucida, improving spermatozoa motility, and protecting spermatozoa DNA integrity. Humans with asthenospermia were treated with L-carnitine and L-acetylcarnitine (32). Furthermore, a metabolomic analysis of bull sperm revealed that high fertility bulls had higher levels of acetylcarnitine in their seminal plasma (33). Carnitine had been identified as a possible SP-biomarker for poor sperm quality (34). In a metabolomic study of boar seminal plasma, the concentration of carnitine in it was found to be negatively correlated with the motility and storage capacity of boar sperm (15). Although carnitine and acetylcarnitine concentrations were found to be significantly higher in Liang guang Small-spotted pig seminal plasma than in Duroc seminal plasma, these metabolites were observed to be lower in LS than in DS. Furthermore, it had been suggested that the presence of L-acetylcarnitine in spermatozoa is related to sperm motility (35). Sperm undergoes significant metabolic and energetic changes when stored at 17 °C for an extended period of time (36). Monosaccharides provide energy to mammalian spermatozoa through glycolysis or a combination of glycolysis and the mitochondrial tricarboxylic acid (TCA) cycle (37). Seminal plasma contains a large number of energy substrates, and different combinations of energy substrate ratios can affect sperm storage capacity and quality in boars at 17 °C (38).

A large number of energy substrates, such as D-Sorbitol, D-Fructose, and citrate, were significant difference in DSP vs. LSP in the current study. According to KEGG enrichment analysis, these metabolites were involved in energy metabolic pathways such as the citrate cycle (TCA cycle), pentose phosphate pathway, carbon metabolism, galactose metabolism, Fructose and mannose metabolism (Figure 6). Previous research had shown that both glycolysis and pentose phosphate pathway were required for goat sperm viability during semen storage, especially the pentose phosphate pathway were more important (39).

In this study, significant differences were found in the energy substrates of seminal plasma between two pig breeds. To the best of our knowledge, there were differences in energy substrates in seminal plasma between species that were related to the spermatozoa's energy metabolic pathways (40, 41). In the seminal plasma of Duroc and Liang guang Small-spotted pigs, a total of 40 different metabolites were discovered. The majority of differential metabolites, such as succinic acid, were energy metabolites. The addition of succinate during sperm energetic acquisition had been shown to maintain a high mitochondrial membrane potential (42). Organic osmolytes such as inositol, D-sorbitol, D-glutamic acid, and L-carnitine were abundant in epididymal fluid, and these organic osmolytes were thought to aid sperm adaptation to the osmotic pressure of the female reproductive tract (43). According to studies (44), inositol increased sperm motility, oxygen consumption, and protected sperm from oxidative DNA damage. In addition, inositol had been shown to improve sperm motility in oligoasthenospermia patients *in vitro* (45). When commercial-grade swine semen for AI was stored at 17 °C, the sperm quality gradually degraded, indicating differences in their sperm recovery ability to fluid storage (15).

Differential metabolites between LS and DS, such as glycerol 3-phosphate, acetylcholine, choline, and glycerophosphocholine, were found to be primarily enriched in glycerophospholipid metabolism in this study. Phospholipids, which made up the majority of the sperm plasma membrane, were critical for spermatozoa's membrane stability. A series of fertilization processes, such as energization and acrosome reactions, had accompanied changes in the lipid composition of the sperm plasma membrane (46). When compared to zebu bulls, crossbred bull spermatozoa contained higher concentrations of phospholipid metabolites and showed higher phospholipid glycerol metabolic pathways, which could be related to the crossbred bull spermatozoa's early acquisition and fertilization ability (47). Duroc spermatozoa had higher levels of phospholipid glycerol metabolism in this study, which could be related to the fact that Duroc spermatozoa had a better ability to recover when stored at 17 °C.

## 5. Conclusion

The semen quality of Liang guang Small-spotted pig and Duroc were analyzed comprehensively, and it was found that the semen storage capacity of the Liang guang Small-spotted pig was weaker than that of the Duroc. Further, metabolomic analysis of seminal plasma and spermatozoa was performed by using UPLC Q-TOF/MS technology in Liang guang Small-spotted pig and Duroc, respectively. The findings demonstrated that 40 metabolites, mostly related to energy metabolism substrates and antioxidant capacity, changed between LSP and DSP, while 17 metabolisms, primarily related to glycerophospholipid metabolism, differed between LS and DS. The analysis revealed that the composition of seminal plasma and spermatozoa and the findings presented here might improve the understanding in spermatozoa and seminal plasma metabolic pathways and the potential for biomarkers. Metabolites involved in energy metabolism, antioxidant capacity and glycerophospholipid metabolism may be key to the poor sperm storage capacity.

## Data availability statement

The original contributions presented in the study are included in the article/Supplementary material, further inquiries can be directed to the corresponding authors.

## Ethics statement

The animal study was reviewed and approved by Animal Protection and Ethics Committee and Use Committee of Foshan University.

## Author contributions

JS and YZ designed the study. JS, KL, JQ, and YS performed, collected data from experiment, and analyzed data. JS and ZL wrote the manuscript. ZL and SE-A revised the manuscript. JZ provided the samples. All authors read and approved the final manuscript.
